# Patients’ experiences managing cardiovascular disease and risk factors in prison

**DOI:** 10.1186/s40352-016-0035-9

**Published:** 2016-04-02

**Authors:** Emily H. Thomas, Emily A. Wang, Leslie A. Curry, Peggy G. Chen

**Affiliations:** 1 Department of Internal Medicine, University of California – San Francisco, San Francisco General Hospital, 1001 Potrero Avenue, Building 10, 3rd Floor, Ward 13, San Francisco, CA 94110 USA; 2 Department of Internal Medicine, Yale University School of Medicine, P.O. Box 208056, 333 Cedar Street, New Haven, CT 06520 USA; 3 Department of Health Policy and Administration, Yale School of Public Health, P.O. Box 208034, 60 College Street, New Haven, CT 06520 USA; 4 RAND Corporation, 1776 Main Street, Santa Monica, CA 90407 USA

**Keywords:** Prison, Cardiovascular disease, Incarceration, Chronic disease management, Self-management support, Self-care

## Abstract

**Background:**

Despite greater risk of cardiovascular disease (CVD) mortality in patients with a history of incarceration, little is known about how prisons manage CVD risk factors (CVD-RF) to mitigate this risk.

**Methods:**

We conducted in-depth interviews with individuals with CVD-RF who had been recently released from prison (*n* = 26). These individuals were recruited through community flyers and a primary care clinic in Connecticut. Using a grounded theory approach and the constant comparative method, we inductively generated themes about CVD-RF care in prisons. Data collection and analysis occurred iteratively to refine and unify emerging themes.

**Results:**

Four themes emerged about care in prison: (1) Participants perceive that their CVD-RFs are managed through acute, rather than chronic, care processes; (2) Prison providers’ multiple correctional and medical roles can undermine patient-centered care; (3) Informal support systems can enhance CVD-RF self-management education and skills; and (4) The trade-off between prisoner security and patient autonomy influences opportunities for self-management.

**Conclusions:**

Patients develop self-management skills through complex processes that may be compromised by the influence of correctional policies on medical care. Our findings support interventions to engage peers, medical providers, care delivery systems, and correctional staff in cultivating effective self-management strategies tailored to prison settings.

## Background

More than two million people are incarcerated in U.S. prisons and jails, or one in every 100 adults (One in 31: The long reach of American corrections 2009). Correctional populations suffer disproportionately from chronic conditions, like hypertension, hyperlipidemia, diabetes, and obesity (Binswanger et al. 2009; Wilper et al. 2009) that promote cardiovascular disease (CVD). More specifically, incarceration is associated with many risk factors for poor cardiovascular health, including low socioeconomic status (Kaplan and Keil 1993), poor access to health care (Wang et al. 2009), illicit drug use (Fazel et al. 2006), and high smoking rates (Conklin et al. 2000). CVD is the most common cause of death amongst inmates (Noonan and Ginder 2013), and mortality due to CVD is 2 times higher among patients released from prison, as compared with peers who have never been incarcerated (Binswanger et al. 2007).

In response to the burden of chronic conditions in prisoners, the National Commission on Correctional Health Care issued guidelines, incorporating elements of the Chronic Care Model (CCM) (Wagner et al. 1996), for management of hypertension and diabetes (Guidance for Disease Management in Correctional Settings 2014). Reforming chronic care delivery using the CCM has been shown in community settings to improve CVD risk factors (CVD-RF) outcomes (Piatt et al. 2006; Coleman et al. 2009; Lewanczuk 2008) and may reduce CVD progression (Vargas et al. 2007). Yet implementation of the CCM depends critically on fostering patients’ self-management skills (Bodenheimer et al. 2002), namely the daily tasks that patients undertake outside of the clinical setting to effectively care for their conditions, including managing their diet, exercise, and medications.

Despite worse CVD outcomes among prisoners, to date no studies have assessed chronic care practices for CVD-RF in US prisons (Donahue 2014) or how these practices conform to correctional chronic care guidelines, like the ones that have been adopted in Connecticut (Chronic Diseases Services 2003). A few studies have focused on CVD prevention through wellness and exercise interventions in well patients (Khavjou et al. 2007), but none have explored how correctional settings can foster self-management practices in patients with CVD-RF. The correctional health literature, in fact, suggests that patient centered care practices, like self-management, will be difficult to implement in prisons due to security constraints on patient autonomy (Condon et al. 2007; Woodall et al. 2014), poor access to care (Harner and Riley 2013a, 2013b), strained relationships with medical providers (Stoller 2003; Young 2000), and lack of education and programming (Loeb et al. 2007). We elicited patients’ perspectives about their care for CVD-RF to explore how correctional systems support the development of patients’ knowledge and skills for CVD-RF self-management. These findings may guide future interventions to effectively tailor chronic care delivery in prison, particularly given the security constraints inherent to this setting.

## Methods

We conducted in-depth interviews with a purposeful sample of men and women recently released from prison. We employed qualitative methods because the assessment of how health care delivery systems support patient self-management of CVD-RF is a multifaceted and complex process. To our knowledge, this is the first study to qualitatively explore these in-depth processes in recently-released patients.

### Study population

Participants were eligible if they had been released from prison within 6 months; had been diagnosed with CVD or CVD-RFs, including diabetes mellitus (type 1 or type 2), hypertension, hyperlipidemia, or obesity (BMI >30); spoke English; and planned to remain in the study area for 12 months, as these patients were engaged in a larger study to assess their longitudinal CVD-RF outcomes following release from prison. Tobacco use was not included as a CVD-RF, as smoking is prohibited in prisons where this study was conducted. We confirmed patients’ stated disease by chart review.

### Study protocol

Recruitment was multi-pronged and included direct engagement at a primary care clinic for patients with a history of incarceration, participant word of mouth, and flyer distribution at local re-entry organizations. Participants received a $50 gift card for study participation. We used a purposeful sampling strategy to capture diverse perspectives from key groups of interest (gender, race/ethnicity, disease status) (Sandelowski 1995). We over-sampled women, compared to proportion of women imprisoned, to more fully characterize the unique conditions in women’s-only facilities. Of the 27 participants asked to participate in qualitative interviews, only one declined.

Two members of our research team (EHT and EAW) led semi-structured interviews using a standardized interview guide (Table 1) that included open-ended questions to elucidate how prison facilitated or constrained management of CVD-RFs (Lofland and Lofland 1971). The interviewers introduced themselves as healthcare providers and correctional health researchers. All participants were interviewed face-to-face in individual rooms in a primary care clinic. In addition, we asked participants to reflect on how they managed their CVD-RFs in the community upon release. The interview guide included non-standardized probes to provide clarification of the emerging concepts in these interviews (Patton 1990). Interviews were recorded, professionally transcribed, and reviewed for accuracy. The interviews averaged 42 min (range 12–71 min).

**Table 1:** Interview Guide

Tell me about when you were diagnosed with X^a^
What is it like to have your chronic condition in prison?
What made it easy to manage your chronic condition in prison?
What made it hard to manage your chronic condition in prison?
In prison, what personal strategies did you develop to take care of X?
What makes it easy or hard to manage X now that you have been released from prison?

*X* ^a^diabetes, high blood pressure, high cholesterol, obesity, or heart disease

### Data analysis

Our research team was composed of individuals with content and methods expertise, including a medical student with experience caring for correctional populations (EHT), an internist with expertise in the care and research of corrections populations (EAW), a pediatrician with expertise in qualitative methods (PGC), and a health service researcher with expertise in health care quality improvement (LAC). Three members of our research team (EHT, EAW, and PGC) met regularly to analyze interviews (Curry et al. 2012). We initially reviewed five transcripts to develop a preliminary coding structure through inductive coding in accordance with grounded theory, an approach to qualitative analysis that allows themes to emerge inductively from participants rather than from the preconceptions of the researchers (Corbin and Strauss 1990; Glaser and Strauss 1967). A fourth author (LAC) reviewed these transcripts and the preliminary coding structure to assess comprehensiveness and properties of emerging codes.

After developing a preliminary code structure, we coded the first six transcripts independently, meeting weekly to negotiate consensus and refine our code structure using constant comparative analysis (Bradley et al. 2007). This iterative process refined the coding structure to clarify extant themes and introduce new themes as they emerged from the data. We maintained a thorough audit trail, adding refinements to our code structure and eliminating or consolidating codes where needed (Sbaraini et al. 2011). We reached thematic saturation while iteratively coding the remaining 20 transcripts. Our final code structures included nine distinct codes, each with discrete sub-codes to capture a broad range of experiences in prison and upon release (Table 2).

**Table 2:** Main Concepts related to the Care of CVD-RF in Patients with a History of Incarceration

The Role of Correctional Institutional Control in patient’s health
• Institutional policies that influence health behaviors or coping
The Role of Post-institutional consequences in patient’s health
• Direct or indirect consequences of incarceration that influence health behaviors or coping
The Role of Individual Agency in patient’s health
• Individual choices that influence health behaviors or coping
The Role of Care Delivery in patient’s health
• Barriers to care, patient education, perceptions of care, tailored care, and fees
Chronic Disease Management
• Medication administration, diet, exercise, self-monitoring, and multi-morbidity
The Role of Interpersonal interactions in patients’ health
• Interactions with other prisoners, prison staff, medical staff, family, non-prisoner peers, criminal justice staff, and an absence of relationships
The Role of Group Membership in patients’ health
• Religious status, financial status, disease status, prison employment, and length in prison influence health behaviors
Comparisons between locations of chronic disease management
• Prison and the community, prison and other prisons, and temporally between prisons
Desires for additional supports

EHT then systematically applied the final codes to all transcripts. We used qualitative analysis software (ATLAS.ti 5.0; Scientific Software Development, Berlin, Germany) to facilitate data organization and retrieval for the purposes of data analysis. This study was part of a larger pilot study of individuals with CVD-RF and was approved by the Human Investigation Committee at Yale University, the Connecticut Department of Corrections Research and Advisory Committee, and the United States Office for Human Research Protections.

## Results

### Demographics

Among the 26 participants, the average time to enrollment after release from prison was 76 days. We sampled purposively to achieve a diverse range of perspectives about CVD-RF management in prison (Table 3). Twenty-five participants reported that they saw a medical provider and were prescribed medications in prison.

**Table 3:** Participant characteristics

Key characteristics	(*n* = 26)
Mean age, years (range)	43 (23–61)
Male, *n* (%)	17 (65)
Never Married, *n* (%)	13 (50)
Less than high school education, *n* (%)	6 (23)
Race/ethnicity, *n* (%)	
Black	16 (61)
White	8 (31)
Hispanic	2 (8)
Cardiovascular Disease Risk Factors (CVD-RF)^a^	
Hypertension	16
Hyperlipidemia	14
Diabetes Mellitus	13
CVD	2
Obesity (BMI >30)	18
Mean Number of CVD-RF (range)	2.4 (1–5)
Incarceration History	
Mean length of most recent incarceration, days (range)	858 (77–3666)
Time to enrollment from release, days (range)	76 (3–181)
Health Care Parameters	*n* (%)
Had a routine medical provider prior to incarceration	21 (81)
Saw medical provider in prison	25 (96)
Prescribed medication in prison	25 (96)
New Diagnoses in Prison	
Any chronic condition	18 (69)
Hypertension	8 (31)
Diabetes Mellitus	5 (19)
Medium or High Health Literacy^b^	17 (71)

^a^These numbers represent the number of participants with these conditions. Seventeen participants had more than one CVD-RF. ^b^Only 24 participants completed the health literacy survey

### Themes

A number of themes about CVD-RF management in prison and upon release emerged in our study. This paper presents the four themes specifically related to chronic care delivery and its influence on self-management practices in prison: (1) Participants perceive that their CVD-RFs are managed through acute, rather than chronic, care processes; (2) Prison providers’ multiple correctional and medical roles can undermine patient-centered care; (3) Informal support systems can enhance CVD-RF self-management education and skills; and (4) The trade-off between prisoner security and patient autonomy influences opportunities for self-management.

#### Participants perceive that their CVD-RFs are managed through acute, rather than chronic, care processes

Participants reported routine screening and treatment of CVD-RFs during their intake medical visit at the beginning of their prison term; however, they expressed many barriers to continued, comprehensive care for their CVD-RF after intake. One patient suggested that the prison clinics were overburdened, and therefore patients had limited opportunities to access medical providers for disease monitoring or education:

“The medical units … need more help…[T]hey need people in there to focus and teach them about their disease… You’re supposed to have checkups… They don’t got no “open door”, nothing in medical [clinics]. They don’t have no diabetes meetings, no blood pressure meetings, health seminars and stuff like that” (44 year-old black man).

Medical care in prison was largely organized by a triage system, where medical providers saw patients based on medical acuity in urgent care visits called “sick call.” One participant described this process as similar to the “emergency department.” Once diagnosed with a CVD-RF, participants relied on the “sick call” system for medical care. One participant described how the triage process contributed to delays in care, even for complications from his anti-hypertensive medication:

“You have to write a medical request… And usually the doctor sees… the people who need the emergency treatment… you’re not that much of a priority to him as someone who just came in that needs to be seen, because he’s got… at least 100 to 200 guys coming in daily… you just keep… writing medical request after medical request, and eventually they’ll see you” (48 year-old black man).

In addition to the procedural barriers to seeking a medical provider in prison, some participants reported fees for “sick call.” This co-pay deterred patients, especially those with limited resources, from seeking education about a new diagnosis:

“If you go to sick call, you have to pay three dollars every time you go there. The only money… that I had, was from working, and I made 75 cents a day. So to ask a question that’s going to cost me … 4 days pay” (38 year-old white man).

Conversely, several participants had positive experiences with sick call, describing the system as “easy.” Once a patient placed a medical request, “9 times out of 10 they’ll get back to you the next day… they really did follow-up… if you’ve got a chronic disease such as diabetes…[they’d] see that your needs were met” (48 year-old black man). These divergent perspectives reflected the diversity of care delivery practices across prisons even in one state.

#### Prison providers’ multiple correctional and medical roles can undermine patient-centered care

In prison participants interacted with many members of the medical team, including medical assistants, nurses, and physicians. The relationship between medical providers and patients in prison was multifaceted. Participants often portrayed physicians in roles that were not directly related to medical care. Physicians were gatekeepers to non-medical privileges, such as clearance for desirable bottom bunks or certain jobs. Physicians also gave permissions for disease-based diets that were not readily available to all prisoners. One hypertensive participant remarked that “the doctor has to [give permission]…to get a low-sodium diet…so it takes a process of maybe two weeks” (45 year-old black man).

Further, medical providers enacted correctional roles and sanctioned participants for refusing to take their medications, which had a dual effect of promoting adherence, but also diminishing self-management of adherence behaviors. The sanctions ranged from sending patients to solitary confinement to issuing tickets (resulting in a loss of recreation time, delayed parole, etc.):

“If you don’t get your meds, you get a ticket. So you got no choice but to go get the meds… it’s a routine for me … because you get a ticket if you don’t.” (44 year-old black man)

Medical providers’ correctional roles shaped patients’ perceptions of their medical care. While some providers were described as caring and attentive, others are described as neglectful or suspicious of malingering. Conversely, one participant reflected on how the prison setting shaped medical providers’ perceptions of care provision:

“[Medical providers are] in a hostile environment, so I believe it’s a lot harder and difficult…to deal with people…they’ll look at each individual as the same, instead of looking at each individual differently…” (48 year-old black man).

Furthermore, participants perceived that medical providers had to adhere to a treatment protocol at the expense of individualizing treatment plans. By adding exercise to his medical treatment, one diabetic participant achieved normoglycemia. When he asked his provider to discontinue his medications, the participant reported that his provider replied, “Yeah, you probably did help yourself by losing weight, but you’re still going to take the pill… [I]t’s been ordered for you, so you gotta take it until where it runs out.” (46 year-old white man).

Alternatively, several participants described how medical providers successfully tailored interventions to patients in prison. One woman explained a nutrition intervention:

“[I]t was called Women Overweight group … [the nurse] would lay out everything that was on commissary that was like good for you… [S]he taught us like how to rinse off the peanuts from the salt…[S]he would have a whole class based on like your options… in the chow hall… like what is good for you, how many calories are in that…” (29 year-old woman).

#### Informal support systems can enhance CVD-RF self-management education and skills

Patients actively sought resources from fellow prisoners, family members, and correctional officers to enhance their education and skills. Many participants looked to other prisoners to share books and disease-based education, as in this diabetic participant’s example: “I didn’t know the difference between Lantus and regular NPH…that one was a long-acting and one was a fast-acting…but I found that out from another prisoner.” (48 year-old black man).

Fellow prisoners also contributed by identifying new diagnoses or complications from CVD-RF, as this new-onset diabetic patient explained, “I passed out coming out of the chow hall, and honestly it was another inmate who suggested that when I go to the medical unit that they… check my sugars” (51 year-old black woman). Certain prisons recognized the vital role that fellow prisoners could play in health promotion and established interventions to train prisoners to become certified nursing assistants (CNAs). One participant remarked that “…the CNAs that were inmates, they were great… they were more caring than those nurses ever were, and these were men caring for men…” (48 year-old black man).

Beyond peers, participants identified family members and correctional officers as educators. One woman newly diagnosed with hypertension reported that “[t]he only… education I did have was from my family because… a lot of people in my family had suffered from high blood pressure” (43 year-old black woman). Participants frequently interacted with correctional officers on the prison units, and participants remarked that correctional officers could support their CVD-RF self management outside of the medical unit. A diabetic man noted that his “[correctional] officer… used to be a nurse. [S]he would ask me every day how I was doing, how I’m feeling, how my sugar was. She was the one who explained to me what ketones were. Not the medical staff” (38 year-old white man).

#### The trade-off between prisoner security and patient autonomy influences opportunities for self-management

The policies of each medical unit in prison dictated how fully participants engaged in self-management practices. These policies exposed the trade-offs between prisoner security and patient autonomy, which were reflected in opportunities for participants to self-monitor their conditions, administer medications, and manage complications. These opportunities were often conditional and varied by correctional facility, medication type, and provider discretion. In some facilities, diabetic patients were taught to use glucometers, but no diabetic patient in our study reported self-injecting insulin. One participant noted the repercussions of this lack of training:

“One of the major problems I had was, obviously they don’t give you needles in prison… so I never learned how to inject myself. They do give you like a crash course the day before you leave, but… they never gave me information on how much insulin I’m supposed to use compared to what my sugar is. I have insulin at home now and never used it, even when my sugar was high, because I don’t know how to do it…” (38 year-old white man).

Prisons differed in their processes of administering lower risk medications, including medications that do not require injections. In some prisons, patients received their medications at clinic, where the staff “check your mouth…and make sure you swallow the pill….” (54 year-old black man). In contrast, other prisons allowed patients to keep medications in their cells, or “keep on person” (KOP), often dispensed “on a strip [or] a bulkie,” individually wrapped, daily medication packets. Patients endorsed that KOP medications gave them the opportunity to practice self-administrating medication and reinforce adherence behaviors. Some participants reported that medical providers only delegated these self-management practices to patients after a supervisory period:

“In the beginning… they don’t know you if you’re responsible enough to take your meds… [After] 3 months … they finally called me down for my physical …and the doctor was like, “Well now that I’ve seen you, I can tell them that you can have your medication on you” (39 year old white woman).

Despite many restrictions on self-management, patients invented strategies to manage their CVD-RFs. Patients learned to manage hypoglycemic complications that frequently occurred at night, when access to correctional officers or medical providers was limited, by smuggling sugar packets from the cafeteria to the unit, or as this patient, who could afford commissary food, described:

“I wouldn’t even try to go to medical. I would just go in my locker and eat … snickers, little debbie cakes and stuff that… I keep for like if I ever be in a predicament… I’d probably eat two of them just to get my sugar up there real fast” (25 year-old black man).

## Discussion

Our findings highlight the potential for prisons to strengthen patient-centered care by reinforcing patients’ CVD-RF self-management. Because prisons offer a stable environment and guaranteed access to care, where vulnerable patients may be screened and treated for many CVD-RF for the first time, they are well-suited to design interventions to teach skills to improve CVD outcomes. Self-management practices are central to CVD-RF control and have recently been incorporated into guidelines for care in prisons (Yusuf et al. 2004; Eckel et al. 2014). Our study participants identified barriers to care in prison, including co-pays, long wait times, and poor patient-provider communication, that previous studies had cited (Condon et al. 2007; Woodall et al. 2014; Harner and Riley 2013a, 2013b; Stoller 2003; Young 2000; Loeb et al. 2007), as well as novel barriers to care, including lack of access to self-management tools, like glucometers and insulin pens. Prisons may overcome these limitations by focusing on three keys areas of CVD-RF care, including access to non-urgent care, provider-based practices, and self-management support.

First, participants’ had limited access to chronic care, and as a result, participants often defaulted to the sick call system for medical issues that were by their nature sub-acute, like education for a new diagnosis or medication complications. These practices may have interesting implications for patients’ care-seeking behaviors upon release. Studies have demonstrated that patients released from prison have higher utilization of acute care services for conditions that are preventable with engagement in primary care (Wang et al. 2013). Our qualitative findings suggest that these acute care-seeking patterns may be reinforced in prison, and these behaviors warrant further investigation. Additionally, co-payments for medical care, which have been shown to contribute to delays and medical complications in prison (Methicillin-resistant Staphylococcus aureus infections in correctional facilities---Georgia, California, and Texas, 2001–2003 2003), further discouraged participants from seeking care for CVD-RFs. By scheduling visits free of charge, prisons may shift the CVD-RF treatment paradigm from an acute care model to a comprehensive chronic care model. These shifts have the potential to not only improve treatment outcomes, but also be cost-effective, as demonstrated in the Texas prison system (Raimer and Stobo 2004).

Second, participants described how medical providers’ dual medical and correctional role has ethical and practical consequences. Dual loyalty refers to the ethical conflicts that providers negotiate in caring for patients and supporting correctional operations (MacDonald et al. 2013; Pont et al. 2012; Willmott 1997). Our study demonstrated that these provider practices, like being a gatekeeper for special diets or punishing patients for medication non-adherence, practically impacted care provision too. Patient-provider relationships were at times contentious and undermined productive partnerships critical to building self-management skills (Holman and Lorig 2000). Additionally participants perceived that providers were unable or unwilling to tailor their care to specific patients. By enabling providers to focus solely on healthcare provision and discouraging participation in punitive correctional policies, providers may build relationships that support patient self-management.

Third, participants’ self-care was subject to correctional oversight, which impaired self-management, self-monitoring, and coping strategies for CVD-RF. Experts estimate that 95 % of care for chronic conditions in the community occurs outside of the clinic (Funnell 2000), yet the controlled setting of prison detracted from patients’ capacity for self-care. Several studies, however, have shown that specific aspects of self-management are feasible and effective in prison. A randomized controlled trial demonstrated that adherence to self-administered HIV medications in prison is comparable to that administered in medication line (White et al. 2015). Self-administered medications also promote patient confidentiality, are more tolerable to patients, and may potentially prepare patients for disease management upon release (Roberson et al. 2009; Hassan et al. 2012; Rieder et al. 2013). As our participants suggested, peer-led education may improve self-management behaviors for CVD-RFs, as has been shown for HIV prevention in prison and upon release (Devilly et al. 2005). By studying and implementing interventions that extend self-care responsibilities to patients, including self-administering insulin, scheduling medical visits to support these practices, and formalizing peer-led education, prisons may implement systems-based approaches to facilitate self-management practices for CVD-RF.

Overall participants voiced that correctional policies and security concerns limited their access to care, interactions with providers, and self-management practices. Divergent accounts suggested that chronic care was readily available; providers were responsive to follow-up; and correctional policies enabled patients to establish routines to adhere to their medication regimens. Participants approved of peer-based care and group education sessions that tailored dietary recommendations to prison. These experiences suggest that many prisons are already implementing strategies to support CVD-RF self-management, and these practices merit further evaluation.

Our findings should be interpreted in light of several limitations. First, we interviewed participants released from a single state’s prisons; therefore our findings may not be transferable to all prisons. This study, however, may guide for potential ways to improve CVD-RF care in prison settings. Second, participants were interviewed within six months from release, and their accounts may be subject to recall bias. Third, most participants were engaged in primary care, had relatively high health literacy, and planned to live in Connecticut for at least 12 months following release (Mallik-Kane and Visher 2008). These unique, stabilizing factors may limit the applicability of the experiences in our sample to other correctional populations. Fourth, members of our research team directly provided healthcare to some study participants. Although this relationship may perpetuate social desirability bias, the familiarity may also be an asset to framing discussions about patients’ healthcare experiences. Finally, our coding team was composed of Asian American and white women from an academic medical center. We were responsive to the fact that our backgrounds were distinctly different than these participants by engaging in reflexivity at our weekly coding meetings to explore and establish distance from our preconceptions in our coding interpretations (Malterud 2001).

## Conclusions

An estimated 95 % of prisoners are released back into the community (The Health Status of Soon-To-Be-Released Inmates 2002), where many encounter poor access to care, medication discontinuities, and the stress associated with transitioning home from prison (Baillargeon et al. 2009; Mallik-Kane and Visher 2008). Prisons may be able to mitigate these risks by fostering self-management practices that patients continue to enact in the community upon release. Our findings point to important areas for future interventions and research to develop effective self-care for CVD-RF in prison. By evaluating the effectiveness and safety of these interventions in prison and upon release, correctional institutions may demonstrate the potential for CVD-RF self-management to improve CVD outcomes, particularly upon re-entry where the risks from CVD mortality are greatest (Binswanger et al. 2007).

## Abbreviations

CVD: cardiovascular disease
CVD-RF: cardiovascular disease risk factors
CNA: certified nursing assistant
KOP: keep on person
